# Promiscuity of Peptides Presented in HLA-DP Molecules from Different Immunogenicity Groups Is Associated With T-Cell Cross-Reactivity

**DOI:** 10.3389/fimmu.2022.831822

**Published:** 2022-02-16

**Authors:** Aicha Laghmouchi, Michel G. D. Kester, Conny Hoogstraten, Lois Hageman, Wendy de Klerk, Wesley Huisman, Eva A. S. Koster, Arnoud H. de Ru, Peter van Balen, Sebastian Klobuch, Peter A. van Veelen, J. H. Frederik Falkenburg, Inge Jedema

**Affiliations:** ^1^Department of Hematology, Leiden University Medical Center, Leiden, Netherlands; ^2^Center for Proteomics and Metabolomics, Leiden University Medical Center, Leiden, Netherlands

**Keywords:** AlloSCT, HLA-DP, peptidome, DPC-classification, CD4 T-cell clones, cross-reactivity

## Abstract

In the context of HLA-DP-mismatched allogeneic stem cell transplantation, mismatched HLA-DP alleles can provoke profound allo-HLA-DP-specific immune responses from the donor T-cell repertoire leading to graft-versus-leukemia effect and/or graft-versus-host disease in the patient. The magnitude of allo-HLA-DP-specific immune responses has been shown to depend on the specific HLA-DP disparity between donor and patient and the immunogenicity of the mismatched HLA-DP allele(s). HLA-DP peptidome clustering (DPC) was developed to classify the HLA-DP molecules based on similarities and differences in their peptide-binding motifs. To investigate a possible categorization of HLA-DP molecules based on overlap of presented peptides, we identified and compared the peptidomes of the thirteen most frequently expressed HLA-DP molecules. Our categorization based on shared peptides was in line with the DPC classification. We found that the HLA-DP molecules within the previously defined groups DPC-1 or DPC-3 shared the largest numbers of presented peptides. However, the HLA-DP molecules in DPC-2 segregated into two subgroups based on the overlap in presented peptides. Besides overlap in presented peptides within the DPC groups, a substantial number of peptides was also found to be shared between HLA-DP molecules from different DPC groups, especially for groups DPC-1 and -2. The functional relevance of these findings was illustrated by demonstration of cross-reactivity of allo-HLA-DP-reactive T-cell clones not only against HLA-DP molecules within one DPC group, but also across different DPC groups. The promiscuity of peptides presented in various HLA-DP molecules and the cross-reactivity against different HLA-DP molecules demonstrate that these molecules cannot be strictly categorized in immunogenicity groups.

## Introduction

Allogeneic stem cell transplantation (alloSCT) is a curative treatment for patients suffering from hematological malignancies (1–9). Fully human leukocyte antigen (HLA) matched sibling or unrelated donors (URD) are preferred as sources of the stem cell grafts. However, in the unrelated setting, 80% of the donors who are considered to be matched for HLA class-I and HLA class-II, are still mismatched for one or two HLA-DP alleles (10/10 match) (10–16). The mismatched HLA-DP allele(s) can induce potent allo-HLA-DP-specific immune responses from the donor T-cell repertoire resulting in graft-versus-leukemia (GvL) effect and/or graft-versus-host disease (GvHD) (17–22). Allo-HLA-reactive T cells are present within all normal T-cell repertoires, since during thymic selection, T cells capable of recognizing peptides in the context of allogeneic HLA molecules are not deleted as they do not encounter these foreign HLA molecules during T-cell development (23, 24). T cells capable of recognizing non-self-peptides in the context of self-HLA have been found to harbor the potential to cross-react with peptides presented in the context of non-self HLA (25–28).

In earlier studies it was shown that allo-HLA-restricted reactivity is determined by both the HLA polymorphism and the presented peptide (29, 30). In the case of HLA-DP molecules it was suggested that the amino acid variability of the peptide-binding groove determined alloreactivity (31, 32). The *in vitro* analyses of allo-HLA-DP-reactive T cells and the polymorphisms within the peptide-binding grooves were used to define the functional distance between the HLA-DP molecules as described in the T-cell epitope (TCE) classification. The TCE groups were used to predict the influence of HLA-DP mismatches on clinical outcome after alloSCT (33–36). The functional distance between HLA-DP molecules and their peptide-binding properties were assumed to result in distinct HLA-DP peptidomes. To investigate this hypothesis, we previously dissected the peptidomes of the most frequently expressed HLA-DP molecules (37). The identification of similarities and differences between the peptidomes for the different HLA-DP molecules resulted in a functional hierarchy. The HLA-DP molecules were categorized into three HLA-DP-peptidome clusters (DPC). The peptidomes of HLA-DPB1*09:01, 10:01 and 17:01 and the peptidomes of HLA-DPB1*04:01, 04:02 and 02:01 separated into the two maximally distinct clusters DPC-1 and DPC-3, respectively. The HLA-DP molecules 13:01, 01:01, 03:01 and 06:01 clustered in DPC-2 and shared certain positions in the peptide-binding motifs with DPC-1 or with DPC-3 molecules while other positions were distinct from the HLA-DP molecules in the other groups. The differences and similarities between the HLA-DP molecules indicated that a more gradual categorization would be appropriate (37).

In this study we aimed to further investigate the categorization by identifying individual peptides that were cross-presented by the thirteen most frequently expressed HLA-DP molecules. K562 cells were transduced with the individual HLA-DP alleles and peptide elution experiments were performed using immunoaffinity chromatography and mass spectrometry. Individual peptides that were presented by several different HLA-DP molecules were identified. The results were in line with the DPC classification, as most of the shared peptides were found to be presented by HLA-DP molecules within DPC groups, especially in the case of DPC-1 and DPC-3. However, for DPC-2, the molecules could be better categorized into two distinct subgroups based on the presented peptides. Although the analyses on shared presented peptides resulted in the overall categorization of HLA-DP molecules in four groups, the data also demonstrated overlap in peptides presented in HLA-DP molecules from different DPC groups. The biological implication of these findings was addressed by analyzing the reactivity of allo-HLA-DP-restricted T-cell clones showing cross-reactive recognition patterns against multiple different HLA-DP molecules, even beyond the boundaries of the DPC classification. The promiscuity of peptides presented in various HLA-DP molecules and the cross-reactivity against different HLA-DP molecules, even from different DPC groups, demonstrate that HLA-DP molecules cannot strictly be categorized in immunogenicity groups.

## Materials and Methods

### The Generation of HLA-DP-Expressing K562 Cell Lines

To investigate the peptides which are presented in the thirteen most frequently expressed HLA-DP molecules with more than 1% population frequency in the Netherlands, HLA class I and II–negative human immortalised myelogenous leukemia (K562) cell lines were transduced with individual HLA-DP alleles to obtain thirteen different K562 cell lines. The β-chain and the corresponding α-chain (**Supplementary Table 1**) were introduced using pLZRS vector HLA-DPA1-T2A-HLA-DPB1-IRES-DNGF-R combinations or as separate chains using MP71 DPA1-IRES-GFP and MP71 DPB1-IRES-DGFR vectors. The HLA-DP-expressing K562 were enriched by flow cytometric cell sorting based on expression of the mutated nerve growth factor receptor (DNGF-R) or green fluorescent protein (GFP) marker and subsequently the expression of the correct HLA-DP alleles was confirmed by sequencing (data not shown). The thirteen K562 cell lines were expanded in Iscove’s modified Dulbecco’s medium (IMDM; Lonza) supplemented with 10% heat-inactivated fetal calf serum (FCS; Gibco). The expanded K562 cell lines were cryopreserved for later use in functional T-cell reactivity assays and 2-8 x 10^9^ cells were stored as dry cell pellets at -80˚C for peptide elution experiments (37).

### HLA-DP Immunoaffinity Chromatography

The hybridoma cell line B7/21 was expanded in Corning hybridigo SF medium to produce anti-HLA-DP antibodies (Abs). Protein A Sepharose beads (GE Healthcare) were used to purify the Abs and generate an immunoaffinity column (B7/21–Protein A Sepharose 2.5 mg/ml) (38). The cell pellets of the different HLA-DP-transduced K562 cell lines were lysed in 50 mM Tris–HCl (pH 8), 150 mM NaCl, 5 mM EDTA, and 0.5% ZWITTERGENT 3–12 (Merck) and supplemented with cOmplete Protease Inhibitor (Merck). The supernatant was precleared with CL4B beads (GE Healthcare) and applied to the immunoaffinity column with a flow rate of 2.5 ml/min. The bound peptide-HLA-DP complexes were eluted with 10% acetic acid (Merck) followed by the separation of peptides from the HLA-DP molecules using a 10-kDa membrane (Merck) (37).

### Mass Spectrometry Analysis and Data Processing

The obtained filtrates containing the peptides were analyzed by online C18 nano-high-performance- liquid-chromatography (HPLC) tandem mass spectrometry (MS/MS) with a system consisting of an Easy-nLC 1200 gradient HPLC system (Thermo Fisher Scientific) and an Orbitrap Fusion Lumos Mass Spectrometer (Thermo Fisher Scientific) or an Orbitrap Exploris Mass Spectrometer (Thermo Fisher Scientific). The detailed procedure from injection to elution has been described previously (37). The raw data of the different HLA-DP peptidomes were first converted to peak lists using Proteome Discoverer version 2.2 (Thermo Electron) and submitted to the Uniprot Homo sapiens database (20,410 entries) using Mascot v. 2.2.07 (http://www.matrixscience.com) for protein identification with target decoy for false discovery rate (FDR). Further analysis was done on datasets with High Confident peptides (FDR 1%), Search Engine Rank = 1 and Mascot Ion Score above 10. Mass spectrometry (MS) contaminants such as keratins were excluded from the data. Peptide annotation, grouping and alignment was done in Access, Excel and R. The High Confident K562-HLA-DP datasets were compared for overlapping peptide sequences and analyzed at different levels without and with normalization for peptide amount and length variants of the same epitope presented in HLA-DP. Normalization for peptide amount was done on the High Confident Peptide to Sequence Matches (PSMs) from Proteome Discoverer and Mascot. High abundant peptides have high numbers of PSMs and low abundant only 1 or a few. The range was 1-169 PSMs on sequence level and expressed in % of total within one sample. To exclude the effect of the different length variants of ligands presented in HLA-DP between the samples, a Region Sequence was defined with a fixed start and end position on the 10^th^ amino acids within a protein containing the identified peptide (37). This dataset of sequences with mainly 10, 20 and 30 amino acid lengths was also normalized on numbers of PSMs and on length variants within a Region Sequence. The Network plot to visualize the % of overlapping peptides between the samples was made in R using ggraph.

### The Generation of Allo-HLA-DP-Restricted CD4 T-Cell Clones

Unmanipulated peripheral blood (without previous granulocyte colony-stimulating factor stimulation) was obtained from healthy donors after informed consent. Responder CD4 T cells were isolated by magnetic activated cell sorting (MACS) from peripheral blood mononuclear cells (PBMC) as previously described (39). The CD4 T cells were stimulated with irradiated (25 Gy) HLA-DP-mismatched antigen-presenting cells (APC) in a 10 to 1 responder to stimulator cell ratio in two different *in vitro* T-cell responses targeting HLA-DPB1*01:01 and HLA-DPB1*03:01 or HLA-DPB1*03:01 and HLA-DPB1*04:01, respectively (HLA-DP typing of the responder and stimulator combinations is shown in **Supplementary Table 2**), as described previously (39). The cells were cultured in IMDM containing 10% heat-inactivated human serum (ABOS; Sanquin) supplemented with IL-7 (10 ng/ml, Miltenyi Biotec), IL-15 (0.1 ng/ml, Miltenyi Biotec) and IL-2 (50 IU/ml, Novartis Sandoz). The cell cultures were restimulated at day 14 with irradiated HLA-DP-mismatched APC. At 24-36 hours after restimulation, reactive CD4 T cells were clonally isolated by single cell flow cytometric cell sorting based on CD137 expression using FACSAria (BD Biosciences), as described previously (39). The T-cell clones were expanded for two weeks using allogeneic feeder mixture consisting of IMDM containing 5% heat-inactivated ABOS and 5% FCS supplemented with 5x irradiated (35 Gy) allogeneic feeder cells, 0.5x irradiated (60 Gy) allogeneic Epstein-Barr virus lymphoblastoid cell lines (EBV-LCL), 100 IU/ml IL-2, and 800 ng/ml phytohemagglutinin (PHA-HA16, Oxoid) (39). The clonality of the generated CD4 T-cell clones was confirmed using ARTISAN polymerase chain reaction (PCR), as previously described (40, 41). The complementarity-determining region-3 (CDR3) of the TCRβ chains were identified to confirm the presence of one TCR clonotype in each CD4 T-cell clone.

### The Analysis of Allo-HLA-DP-Restricted Reactivity

CD4 T-cell clones recognizing only stimulator cells expressing the mismatched target HLA-DP allele(s) and not stimulator cells expressing the autologous HLA-DP allele(s) were selected as being allo-HLA-DP reactive, as described previously (39, 42). The reactivity of allo-HLA-DP-reactive T-cell clones against different HLA-DP molecules was analyzed using the panel of HLA-DP-transduced K562 cell lines as stimulator cells at a 1:5 responder to stimulator ratio in IMDM/10% ABOS supplemented with 25 IU/ml IL-2. The supernatants were harvested after overnight incubation and the amounts of interferon-gamma (IFNγ) and interleukin-4 (IL-4) in the supernatants were quantified using standard enzyme-linked immunosorbent assays (ELISA; Sanquin or Invitrogen). Reactivity was defined as at least 2 times higher cytokine production compared to the non-stimulated condition.

## Results

### Promiscuity of Peptides Presented in HLA-DP Alleles

To identify individual peptide sequences presented by different HLA-DP molecules, K562 cell lines were transduced with various HLA-DPB1 and corresponding HLA-DPA1 chains (**Supplementary Table 1**) and the HLA-DP peptidomes were analyzed by immunoaffinity chromatography and mass spectrometry. Identical peptide sequences with various amino acid lengths presented in different HLA-DP molecules were unraveled. To correct for an effect of length variants, peptide sequences were aligned to identify tens of amino-acid long region sequences to include distinct peptides only once in the analyses. Peptides encoded by the gene *itgam* eluted from HLA-DPB1*09:01, HLA-DPB1*05:01 and HLA-DPB1*11:01 are shown as examples of correction for length variants by identifying the region sequences (**Table 1**). The peptidome analyses resulted in the detection of a median of 3131 peptide sequences per HLA-DP molecule (range 2172-4449), including length variants, and a median of 1566 distinct region sequences per HLA-DP molecule (range 1207-2168).

**Table 1:** Examples of ITGAM peptides and their length variants and how the region sequences are identified.

	Protein name	Start	Length	Peptide sequences (aligned)	Region sequences
HLA-DPB1*09:01	ITGAM	571	13	---IAGSKLSPRLQYF	IAGSKLSPRLQYFGQSLSGG
	ITGAM	571	14	---IAGSKLSPRLQYFG	IAGSKLSPRLQYFGQSLSGG
	ITGAM	573	20	------GSKLSPRLQYFGQSLSGGQD	IAGSKLSPRLQYFGQSLSGGQDLTMDGLVD
	ITGAM	574	19	--------SKLSPRLQYFGQSLSGGQD	IAGSKLSPRLQYFGQSLSGGQDLTMDGLVD
	ITGAM	575	18	---------KLSPRLQYFGQSLSGGQD	IAGSKLSPRLQYFGQSLSGGQDLTMDGLVD
	ITGAM	576	14	-----------LSPRLQYFGQSLSG	IAGSKLSPRLQYFGQSLSGG
	ITGAM	576	15	-----------LSPRLQYFGQSLSGG	IAGSKLSPRLQYFGQSLSGG
	ITGAM	576	16	-----------LSPRLQYFGQSLSGGQ	IAGSKLSPRLQYFGQSLSGGQDLTMDGLVD
	ITGAM	576	17	-----------LSPRLQYFGQSLSGGQD	IAGSKLSPRLQYFGQSLSGGQDLTMDGLVD
HLA-DPB1*05:01	ITGAM	371	16	---SYDWAGGVFLYTSKEK	SYDWAGGVFLYTSKEKSTFI
	ITGAM	374	15	--------WAGGVFLYTSKEKST	SYDWAGGVFLYTSKEKSTFI
	ITGAM	374	16	--------WAGGVFLYTSKEKSTF	SYDWAGGVFLYTSKEKSTFI
	ITGAM	375	13	-----------AGGVFLYTSKEKS	SYDWAGGVFLYTSKEKSTFI
	ITGAM	375	14	-----------AGGVFLYTSKEKST	SYDWAGGVFLYTSKEKSTFI
	ITGAM	375	15	-----------AGGVFLYTSKEKSTF	SYDWAGGVFLYTSKEKSTFI
	ITGAM	376	13	-------------GGVFLYTSKEKST	SYDWAGGVFLYTSKEKSTFI
	ITGAM	377	12	---------------GVFLYTSKEKST	SYDWAGGVFLYTSKEKSTFI
	ITGAM	379	10	-------------------FLYTSKEKST	SYDWAGGVFLYTSKEKSTFI
HLA-DPB1*11:01	ITGAM	41	16	---LQGSRVVVGAPQEIVA	LQGSRVVVGAPQEIVAANQR
	ITGAM	41	17	---LQGSRVVVGAPQEIVAA	LQGSRVVVGAPQEIVAANQR
	ITGAM	41	18	---LQGSRVVVGAPQEIVAAN	LQGSRVVVGAPQEIVAANQR
	ITGAM	41	19	---LQGSRVVVGAPQEIVAANQ	LQGSRVVVGAPQEIVAANQR
	ITGAM	41	21	---LQGSRVVVGAPQEIVAANQRG	LQGSRVVVGAPQEIVAANQRGSLYQCDYST
	ITGAM	41	22	---LQGSRVVVGAPQEIVAANQRGS	LQGSRVVVGAPQEIVAANQRGSLYQCDYST
	ITGAM	42	12	-----QGSRVVVGAPQE	LQGSRVVVGAPQEIVAANQR
	ITGAM	42	14	-----QGSRVVVGAPQEIV	LQGSRVVVGAPQEIVAANQR
	ITGAM	42	15	-----QGSRVVVGAPQEIVA	LQGSRVVVGAPQEIVAANQR
	ITGAM	42	16	-----QGSRVVVGAPQEIVAA	LQGSRVVVGAPQEIVAANQR
	ITGAM	43	14	-------GSRVVVGAPQEIVA	LQGSRVVVGAPQEIVAANQR

**Figure 1:**
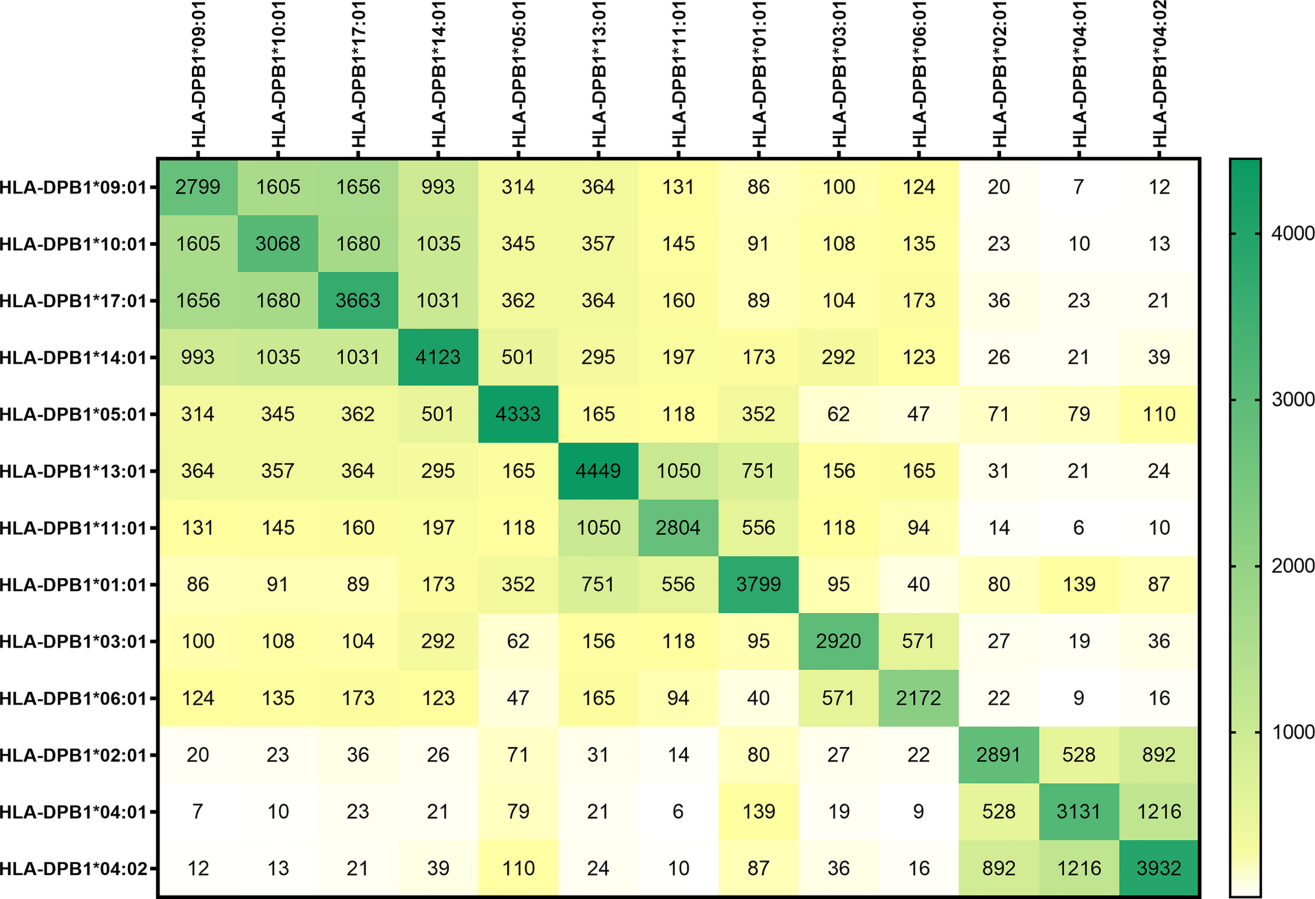
The overlap in peptide sequences presented by the HLA-DP molecules. The peptide sequences presented in the most frequently expressed HLA-DP molecules were unraveled using immunoaffinity chromatography and mass spectrometry. The peptides were quantified, including the length variants, using peptide-to-sequence matching. The absolute number of peptides shared between the HLA-DP molecules are demonstrated. In the dark green boxes at the diagonal, the total number of sequences per HLA-DP molecule are depicted. The numbers of peptides shared between different HLA-DP molecules and the level of overlap in presented peptides is illustrated by the numbers in the heatmap and by coloring of the boxes, with white to yellow indicating no/limited overlap and green indicating relatively abundant overlap.

In **Figure 1**, the peptide sequences shared by the different HLA-DP molecules are shown. In the dark green boxes at the diagonal, the total number of sequences per HLA-DP molecule are depicted. The numbers of peptides shared between different HLA-DP molecules and the level of overlap in presented peptides is illustrated by the numbers in the heatmap and by coloring of the boxes, with white to yellow indicating no/limited overlap and green indicating relatively abundant overlap. The heatmap shows that the level of overlap in presented peptide sequences was most prominent within the two most distinct groups DPC-1 in the upper left corner and DPC-3 in the lower right corner. The differences between groups DPC-1 and DPC-3 are reflected by the minimal overlap in peptide sequences between these groups. Within group DPC-2, the HLA-DP molecules appear to segregate into two subgroups based on the overlap in presented peptides, with subgroup 2A being comprised of HLA-DPB1*13:01, HLA-DPB1*11:01, and HLA-DPB1*01:01 and subgroup 2B containing HLA-DPB1*03:01 and HLA-DPB1*06:01. In **Supplementary Figure 1** we included the peptide-binding motifs of HLA-DPB1*11:01 and the other DPC-2 molecules to demonstrate that the DPC-2 molecules could indeed be separated into two subgroups. The heatmap also shows that HLA-DP molecules from DPC-1 and DPC-2 share substantial numbers of presented peptides. HLA-DPB1*05:01 shows minimal overlap with the HLA-DP molecules from the other DPC-groups. HLA-DP molecules from group DPC-3 shared the lowest numbers of peptides with other molecules. When the length variants were removed, and the data were normalized to region sequences, the same patterns were observed (**Supplementary Figure 2**).

**Figure 2:**
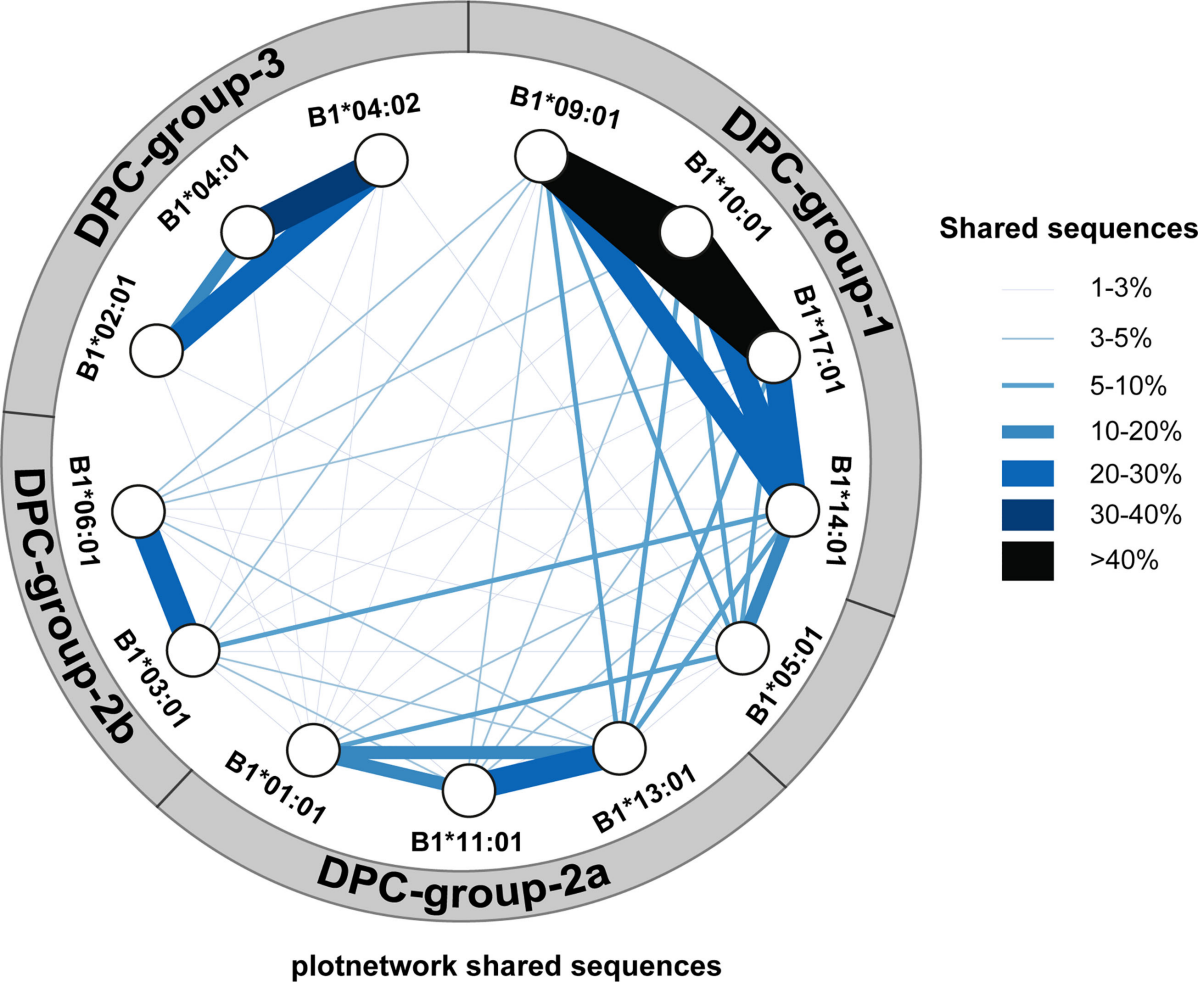
Network of HLA-DP molecules. A network of HLA-DP molecules was generated based on the overlap in presented peptides. The percentage of overlap in presented peptides between the different HLA-DP molecules are illustrated by the thickness and color of the lines as indicated by the legend. The network shows the distinction of four major groups; group DPC-1 consisting of HLA-DPB1*09:01, HLA-DPB1*10:01, HLA-DPB1*17:01 and HLA-DPB1*14:01, group DPC-2A consisting of HLA-DPB1*13:01, HLA-DPB1*11:01 and HLA-DPB1*01:01, group DPC-2B consisting of HLA-DPB1*03:01 and HLA-DPB1*06:01, and finally group DPC-3 consisting of HLA-DPB1*02:01, HLA-DPB1*04:01 and HLA-DPB1*04:01.

To better visualize the similarities in peptides presented in the different HLA-DP molecules, percentages of shared peptides were plotted in a network graph (**Figure 2**). The level of overlap in presented peptides between the different HLA-DP molecules are illustrated by the thickness and color of the lines. Like the heatmap figure, the network plot indicates a distinction of four groups, with HLA-DPB1*05:01 clustered separate from the DPC groups due to very limited overlap in presented peptides with HLA-DP molecules from the DPC groups. The HLA-DP molecules within groups DPC-1 and DPC-3 share a high number of presented peptides as indicated by the thick and black/dark-blue lines. Within group DPC-1 >20% of presented peptides were shared between the HLA-DP molecules and within group DPC-3 the overlap in presented peptides was 10-40% between the HLA-DP molecules. Again, DPC-2 separated into groups DPC-2A and DPC-2B. All of the analyzed HLA-DP molecules could be attributed to one of the four groups in the plot, except for HLA-DPB1*05:01 that only showed limited overlap of peptides (10-20%) with HLA-DPB1*14:01 but minor overlap (<10%) with the other molecules from DPC-1 or from the other DPC groups. This network plot also shows that whereas DPC-3 has hardly any overlap with molecules from other DPC groups, DPC groups 1 and 2 share between 2-10% peptide sequences between the various HLA-DP molecules. The network plot based on region sequences demonstrated similar categorizations and connections (**Supplementary Figure 3**).

### Allo-HLA-DP-Reactive T Cells Can Show Cross-Reactivity Against HLA-DP Molecules Within or Outside DPC Groups

To search for biological relevance of the overlap in peptides presented in different HLA-DP molecules within or outside different DPC groups, the reactivity of several *in vitro* generated allo-HLA-DP-reactive T-cell clones was tested against the panel of HLA-DP-transduced K562 cell lines. The allo-HLA-DP-reactive T-cell clones were derived from two *in vitro* T-cell responses targeting HLA-DPB1*01:01 and HLA-DPB1*03:01 or HLA-DPB1*03:01 and HLA-DPB1*04:01, respectively, as described previously (39). HLA-DP typing of the responder and stimulator combinations is shown in **Supplementary Table 2**. Large numbers of allo-HLA-DP-reactive T-cell clones (170 from response 1 and 440 from response 2) were identified previously (39). In-depth analyses of allo-HLA-DP-directed cross-reactivity using the panel of HLA-DP-transduced K562 cell lines was performed with a selected number of allo-HLA-DP reactive T-cell clones (16 from response 1 and 19 from response 2) with consistent reactivity and sufficient proliferative capacity. The clonality and the diversity of the TCR usage of the allo-HLA-DP-reactive T-cell clones included in this study were confirmed by sequencing their CDR3-beta(β) regions. Based on this analysis 7 unique T-cell clones with their single CDR3-β sequence (3 from response 1 and 4 from response 2) were identified (**Table 2**).

**Table 2:** Overview of the selected T-cell clones with different HLA-DP recognition patterns.

Figure			Reactivity pattern*				#shared peptides~	#shared regions^+^
	Clone name	CDR3 sequence^	DPC-1	DPC-2A	DPC-2B	DPC-3		
**3A**	R2-B055	CASNRQGAYGYTF	–	–	03:01	–	–	–
**3A**	R1-B005	CAWTPRRSHTQYF	–	01:01	–	–	–	–
**3B**	R2-B259	CASSATENRNSPLHF	–	–	–	04:01/04:02	1216	727
**3B**	R1-F040	CASSGGTSGGTEQFF	–	13:01/01:01	–	–	751	474
**3C**	R2-B209	CASGMGVYGYTF	14:01	–	03:01	–	292	207
**3C**	R2-B022	CASSLRLQQLAFF	14:01	–	03:01/06:01	–	91	84
**3C**	R1-H008	CASSLRVREPQHF	14:01	11:01	03:01/06:01	–	29	14

*The reactivity against different HLA-DP molecules, with the target HLA-DP molecules underlined.

^The clonality of the T-cell clones was analyzed by sequencing the complementarity-determining region-3 (CDR3) of the TCRβ chains.

~The shared peptide sequences of the recognized HLA-DP molecules.

^+^The shared region sequences of the recognized HLA-DP molecules.

**Figure 3:**
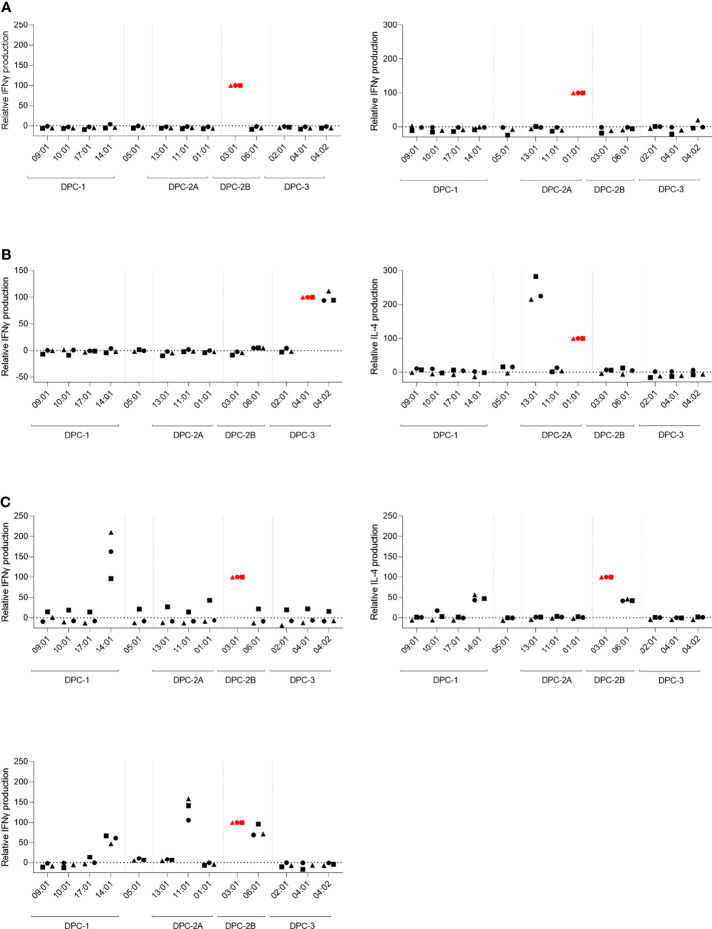
T-cell clones exerting cross-reactivity against HLA-DP molecules from the same or different DPC groups. The relative reactivity of allo-HLA-DP-directed CD4 T-cell clones after overnight stimulation with the panel of HLA-DP-transduced K562 cell lines. The concentration of IFNγ or IL-4 was measured in the supernatants using ELISA. The levels of cytokine production upon cross-reactivity were normalized for the level of cytokine production upon stimulation with the K562 expressing the respective target HLA-DP molecule. **(A)** CD4 T-cell clones with reactivity against single target HLA-DP molecule. **(B)** CD4 T-cell clones with reactivity against the target HLA-DP molecule and cross-reactivity against another HLA-DP molecule within the same DPC group. **(C)** CD4 T-cell clones with cross-reactivity against multiple HLA-DP molecules within the same DPC group as the target HLA-DP molecule, as well as against HLA-DP molecules from another DPC group. The red colored symbols are the measurements after stimulation with the K562 expressing the target HLA-DP molecule. Each symbol represents one experiment.

In response to stimulation of these 7 unique HLA-DP-reactive T-cell clones with the panel of HLA-DP-transduced K562 cell lines, different reactivity patterns were observed. The levels of IFN-gamma/IL-4 production upon cross-reactivity against K562 cells expressing additional allogeneic HLA-DP-molecules were normalized for the level of IFN-gamma/IL-4 production by the T-cell clones in response to stimulation with reactivity against K562 cells expressing the target allogeneic HLA-DP molecule (**Figure 3**). The non-normalized data are shown in **Supplementary Figure 4**. **Figure 3A** shows the T-cell clones with restricted reactivity against only the targeted mismatched HLA-DP molecule (HLA-DPB1*03:01 or HLA-DPB1*01:01) (red symbols). **Figure 3B** shows the T-cell clones that exerted additional cross-reactive recognition against a different HLA-DP molecule within the same DPC group. The observation was made that targeting of HLA-DPB1*04:01 was often associated with cross-reactivity against HLA-DPB1*04:02 (data not shown). In **Figure 3C**, reactivity of three T-cell clones is shown that were found to be cross-reactive against HLA-DP molecules from different DPC groups.

To estimate the possibility that these cross-reactive T-cell clones recognized identical peptides in different HLA-DP molecules, we searched for shared peptides and shared regions in the peptidomes. As depicted in **Table 2**, indeed substantial numbers of shared peptides could be identified in the different HLA-DP molecules recognized by the T-cell clones, although our analyses did not include proof that the same peptide is recognized in these various HLA-DP molecules. The results illustrate that cross-reactivities are not only directed against HLA-DP molecules from the same DPC group, but also against HLA-DP molecules from different DPC groups.

## Discussion

The aim of this study was to investigate a possible categorization of HLA-DP molecules based on identification of individual peptides presented by the thirteen most frequently expressed HLA-DP molecules and to compare this categorization with the previously identified DPC groups (37). K562 cell lines lacking endogenous expression of HLA were transduced with the β-chains and the corresponding α-chains of 13 different HLA-DP alleles. The K562 cell lines were expanded for peptide elution studies to measure their HLA-DP peptidomes using immunochromatography and mass spectrometry. Comparison of the different HLA-DP peptidomes demonstrated an overlap in individual peptides presented by HLA-DP molecules corroborating the previously described DPC classification. The HLA-DP molecules within groups DPC-1 and DPC-3 were found to share the highest numbers of peptides with their group members. For DPC-2, our study showed a segregation into subgroup DPC-2A, comprising HLA-DPB1*13:01, HLA-DPB1*11:01, and HLA-DPB1*01:01, and subgroup 2B, containing HLA-DPB1*03:01 and HLA-DPB1*06:01 based on the overlap in presented peptides in these HLA-DP molecules. Additionally, this study also showed a substantial overlap in presented peptides between various HLA-DP molecules from groups DPC-1 and DPC-2, so even outside the boundaries of the DPC classification. The functional relevance of these findings was illustrated by analyzing allo-HLA-DP-directed T-cell reactivity. We identified allo-HLA-DP-reactive CD4 T-cell clones not only cross-recognizing HLA-DP molecules within the same DPC group, but also across different DPC groups. The promiscuity of peptides presented in various HLA-DP molecules and the cross-reactivity against different HLA-DP molecules demonstrate that HLA-DP molecules cannot strictly be categorized in immunogenicity groups.

Using the HLA negative K562 model transduced with individual HLA-DP molecules instead of for instance EBV-LCL from individuals with different HLA-DP typings rules out the disturbing factor of variation caused by presentation in HLA-DP of individual-specific polymorphisms and peptides derived from HLA-class I and other HLA-class II molecules in our HLA-DP peptidome analyses. Van Balen et al. (2020) previously demonstrated the validity of this K562 model by illustrating identical peptide-binding motifs for HLA-DP molecules in K562 versus EBV-LCL (37).

The clinical outcome of HLA-DP-mismatched alloSCT [and donor lymphocyte infusion (DLI)] is influenced by the magnitude of allo-HLA-DP-specific immune responses, which in turn is determined by the immunogenicity of the mismatched HLA-DP allele(s) and the HLA-DP disparity between donor and patient (33–36). Allo-HLA-DP-reactive immune responses depend on the recognition of non-self HLA-DP/peptide complexes and HLA-DP molecules can strongly vary in the peptide content of their binding grooves (31, 32, 37). In the current study we dissected the promiscuity of individual peptides presented in the HLA-DP molecules from the different DPC groups. The HLA-DP peptidomes were compared by analyzing peptide sequences at two different levels, full-length peptide sequences and region sequences. The individual peptide sequences were obtained from the raw data of the peptide elution study. To obtain region sequences, alignment to a fixed position and normalization for the amount of identified PSMs was performed for length variants presented in HLA-DP. This approach was applied to exclude the effect of different length variants on the numbers of identified peptides between the samples. In this study we focused on the overlap of individual presented peptide sequences (including the length variants) between HLA-DP molecules. However, similar analyses using the regions are shown in **Supplementary Figures 1, 2**, showing similar patterns. In our previous study it was also demonstrated that the identified peptide-binding motifs were not influenced by the overrepresentation of peptides with length variants in comparison with the normalization to region sequences (37).

In our current study the overlap in presented peptides was most obvious between the HLA-DP molecules within the DPC-1 and DPC-3 groups as defined in our previous study. The differences between these two groups, as was previously shown by their highly different peptide-binding motifs, is now also illustrated by the negligible overlap in identical presented peptides between the HLA-DP molecules of these two groups. In addition, based on the promiscuity of individual peptides shown in our current study, the molecules from group DPC-2 can be divided into two sub-groups. Previously, the HLA-DP molecules in group DPC-2 were also found to segregate based on the differences in position 9 of the peptide binding motifs (37). In alignment with our previous study, HLA-DPB1*05:01 was found to be separately clustered from the DPC groups due to a lack of clear overlap in presented peptides. In our previous study, HLA-DPB1*11:01 was not included in the analyses (37). However, in our current study HLA-DPB1*11:01 was found to have clear overlap in presented peptides with the molecules HLA-DPB1*01:01 and HLA*DPB1*13:01 and could therefore be grouped in DPC-2A. The peptide-binding motifs of HLA-DPB1*11:01 and the other DPC-2 molecules also corroborated that the DPC-2 molecules could be separated into two subgroups as based on the overlap in presented peptides, making four groups possibly better fitting in the classification of HLA-DP alleles.

In the HLA-DP-mismatched setting, retrospective studies analyzing the role of HLA-DP-mismatching in clinical outcome after alloSCT have been performed based on categorization of the alleles into the originally defined TCE groups (34, 35). The TCE categorization, initially based on *in vitro* recognition experiments combined with analyses on the amino acid sequence differences compared to HLA-DPB1*09:01, categorized alleles in TCE-1, similar to our DPC-1 group, TCE-2 containing HLA-DPB1*03:01, DPB1*14:01, and DPB1*45:01 and the less clearly defined TCE-3, containing alleles not belonging to either group 1 or 2 (31–33). HLA-DP mismatches within the TCE groups were considered to be permissive, whereas HLA-DP mismatches between TCE groups were defined as being non-permissive. In the case of non-permissive HLA-DP mismatches a significantly increased risk of mortality after unrelated alloSCT was observed (35). Additionally, in the study of Petersdorf et al. it was concluded that not only the HLA-DP mismatch itself but also the differences in expression levels of different HLA-DP alleles would determine clinical outcome. They observed in their transplantation model that GvHD is especially expected to occur in patients with high-expression HLA-DP allele(s) transplanted with donors with low-expression HLA-DP allele(s) (20). We hypothesize that re-analysis of the clinical outcome data based on our now more defined and gradual DPC classification and also taking the differential *in vivo* expression levels of specific HLA-DP alleles into account may lead to a better prediction model for the role of HLA-DP mismatching in alloSCT.

In conclusion, our data show a high promiscuity of peptides presented in HLA-DP molecules within the groups DPC-1 and DPC-3, and a lack of promiscuity between DPC-1 and DPC-3 HLA-DP molecules. DPC group 2 may be better sub-categorized into 2 subgroups. Both DPC-2 subgroups show substantial peptide promiscuity with DPC group 1. The relevance of these findings was confirmed with reactivity patterns of allo-HLA-DP-specific CD4 T-cell clones showing cross-reactivity against HLA-DP molecules within as well as outside DPC groups. The variable degrees of overlap in presented peptides between the HLA-DP molecules indicate that HLA-DP molecules cannot strictly be categorized in immunogenicity groups. The gradual levels of similarities and differences between HLA-DP peptidomes demonstrate that the stringent classification of HLA-DP mismatches in strictly permissive and non-permissive is unlikely to hold true. The classification of HLA-DP-mismatches based on a more gradual range of permissiveness and also taking the differential *in vivo* expression levels of high and low expression HLA-DP alleles into account will help in gaining more insight into the immunological impact of HLA-DP mismatching in allogeneic stem cell transplantation.

## Data Availability Statement

The mass spectrometry proteomics data have been deposited to the ProteomeXchange Consortium via the PRIDE [1] partner repository with the dataset identifier PXD030591 and the CDR3 sequences of the described T-cell clones have been deposited to https://www.ebi.ac.uk/ena/browser/view/PRJEB50041.

## Ethics Statement

Donors and patients had given written informed consent to the storage of biomaterials in the LUMC Biobank, and the use of these materials was approved by the institutional medical ethical committee (protocol number B 16.039). The patients/participants provided their written informed consent to participate in this study.

## Author Contributions

AL, MK, CH, LH, WK, WH, AR, PB, and SK performed experiments and analyzed results. AL, MK, WH, and EK prepared the figures. AL, MK, PV, JF, and IJ designed the research and wrote the paper. All authors contributed to the article and approved the submitted version.

## Funding

The research in this manuscript was financially supported by the Dutch Cancer Society (projects NKB 2008-4263 and UL 2013-5989).

## Conflict of Interest

The authors declare that the research was conducted in the absence of any commercial or financial relationships that could be construed as a potential conflict of interest.

## Publisher’s Note

All claims expressed in this article are solely those of the authors and do not necessarily represent those of their affiliated organizations, or those of the publisher, the editors and the reviewers. Any product that may be evaluated in this article, or claim that may be made by its manufacturer, is not guaranteed or endorsed by the publisher.

## Abbreviations

HLA, Human leukocyte antigen; AlloSCT, Allogeneic stem cell transplantation; URD, Unrelated donor; GvL, Graft-versus-leukemia; GvHD, Graft-versus-host disease; TCE, T-cell epitope; DPC, HLA-DP-peptidome cluster; K562, Human immortalised myelogenous leukemia cell lines; DNGF-R, Mutated nerve growth factor receptor; GFP, Green fluorescent protein; IMDM, Iscove’s modified Dulbecco’s medium; FCS, Fetal calf serum; Abs, Antibodies; HPLC, High-performance- liquid-chromatography; MS/MS, Tandem mass spectrometry; MS, Mass spectrometry; PSMs, Peptide to Sequence Matches; MACS, Magnetic activated cell sorting; PBMC, Peripheral blood mononuclear cells; APC, Antigen-presenting cells; ABOS, Human serum; EBV-LCL, Epstein-Barr virus lymphoblastoid cell lines; PCR, Polymerase chain reaction; CDR, Complementarity-determining region; IFNγ, Interferon-gamma; IL-4, Interleukin-4; ELISA, Enzyme-linked immunosorbent assays.
