# Supplementary material for: Promiscuity of Peptides Presented in HLA-DP Molecules from Different Immunogenicity Groups Is Associated With T-Cell Cross-Reactivity
Source: Front Immunol. 2022 Feb 16;13:831822. doi: 10.3389/fimmu.2022.831822 (PMC8888658; doi:10.3389/fimmu.2022.831822)
Supplement: Supplementary file 1 [file DataSheet_1.pdf]

Supplementary Appendix

Promiscuity of peptides presented in HLA-DP molecules from different immunogenicity groups is associated with T-cell cross-reactivity

Aicha Laghmouchi[1*], Michel G.D. Kester[1], Conny Hoogstraten[1], Lois Hageman[1], Wendy de Klerk[1], Wesley Huisman[1], Eva A.S. Koster[1], Arnoud H. de Ru[2], Peter van Balen[1], Sebastian Klobuch[1], Peter A. van Veelen[2], J.H. Frederik Falkenburg[1] and Inge Jedema[1]

[1]Department of Hematology, Leiden University Medical Center, Leiden, The Netherlands

[2]Center for Proteomics and Metabolomics, Leiden University Medical Center, Leiden, The Netherlands

Supplementary Table 1. HLA-DP alleles transduced in K562 cell lines

	**HLA-DPB1**	**HLA-DPA1**
**DPC-1**	09:01	02:01
	10:01	02:01
	17:01	02:01
	14:01	02:01
	05:01	02:02
**DPC-2**	13:01	02:01
	11:01	02:01
	06:01	01:03
	01:01	02:01
	03:01	01:03
**DPC-3**	02:01	01:03
	04:01	01:03
	04:02	01:03

Supplementary Table 2. HLA-DP typing of the responder/stimulator pairs

Response	Responder		Stimulator	
**1**	DPB1*02:01:02	DPC-3	DPB1*01:01	DPC-2
	DPB1*04:01:01	DPC-3	DPB1*03:01	DPC-2
**2**	DPB1*01:01	DPC-2	DPB1*03:01	DPC-2
	DPB1*02:01:02	DPC-3	DPB1*04:01	DPC-3

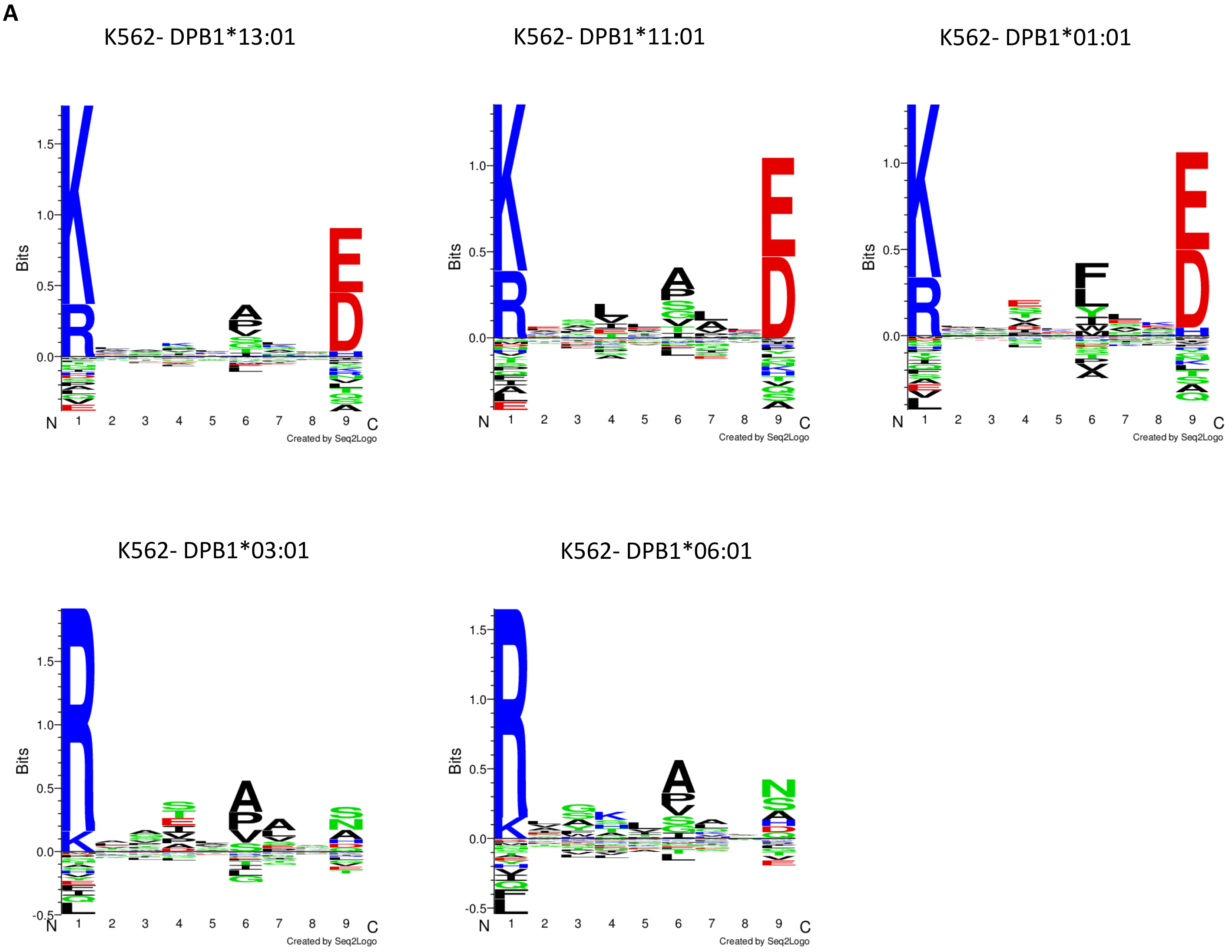

Supplementary Figure 1. Peptide-binding motifs of the DPC-2A and DPC-2B HLA-DP alleles when transduced in K562 cell lines. The Gibbs clustering of eluted peptides was performed as described in our previous study by Van Balen et al. (2020). The identified motifs are demonstrated of peptides presented in both **(A)** DPC-2A and **(B)** DPC-2B HLA-DP molecules. The x-axis depicts the position of amino acids in the peptide, and the y-axis depicts the relative frequency of the amino acids for the different positions in the peptide.

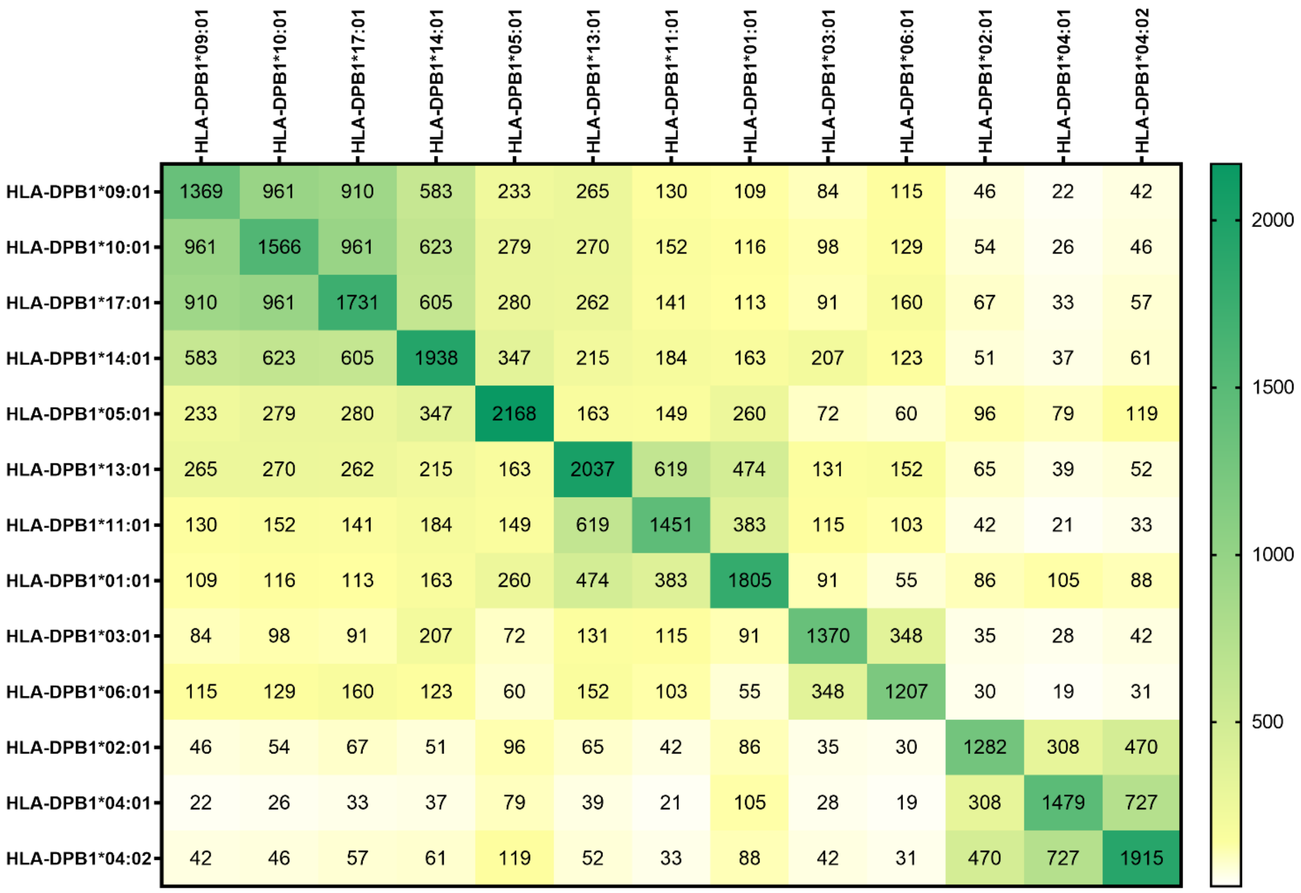

	HLA-DPB1*09:01	HLA-DPB1*10:01	HLA-DPB1*17:01	HLA-DPB1*14:01	HLA-DPB1*05:01	HLA-DPB1*13:01	HLA-DPB1*11:01	HLA-DPB1*01:01	HLA-DPB1*03:01	HLA-DPB1*06:01	HLA-DPB1*02:01	HLA-DPB1*04:01	HLA-DPB1*04:02
HLA-DPB1*09:01	1369	961	910	583	233	265	130	109	84	115	46	22	42
HLA-DPB1*10:01	961	1566	961	623	279	270	152	116	98	129	54	26	46
HLA-DPB1*17:01	910	961	1731	605	280	262	141	113	91	160	67	33	57
HLA-DPB1*14:01	583	623	605	1938	347	215	184	163	207	123	51	37	61
HLA-DPB1*05:01	233	279	280	347	2168	163	149	260	72	60	96	79	119
HLA-DPB1*13:01	265	270	262	215	163	2037	619	474	131	152	65	39	52
HLA-DPB1*11:01	130	152	141	184	149	619	1451	383	115	103	42	21	33
HLA-DPB1*01:01	109	116	113	163	260	474	383	1805	91	55	86	105	88
HLA-DPB1*03:01	84	98	91	207	72	131	115	91	1370	348	35	28	42
HLA-DPB1*06:01	115	129	160	123	60	152	103	55	348	1207	30	19	31
HLA-DPB1*02:01	46	54	67	51	96	65	42	86	35	30	1282	308	470
HLA-DPB1*04:01	22	26	33	37	79	39	21	105	28	19	308	1479	727
HLA-DPB1*04:02	42	46	57	61	119	52	33	88	42	31	470	727	1915

Supplementary Figure 2. The overlap in region sequences presented by the HLA-DP molecules. The peptide sequences presented in the most frequently expressed HLA-DP molecules were unraveled using immunoaffinity chromatography and mass spectrometry. The peptides were quantified, including the length variants, using peptide-to-sequence matching. The length variants of peptide sequences were aligned to identify region sequences (10-30 amino acids depending on the length variants found) to include each distinct peptide sequence only once in the quantifications. The absolute numbers of region sequences shared between the HLA-DP molecules are demonstrated in this figure. The cells are colored according to the number of region sequences from low to high, in the colors white, yellow to green.

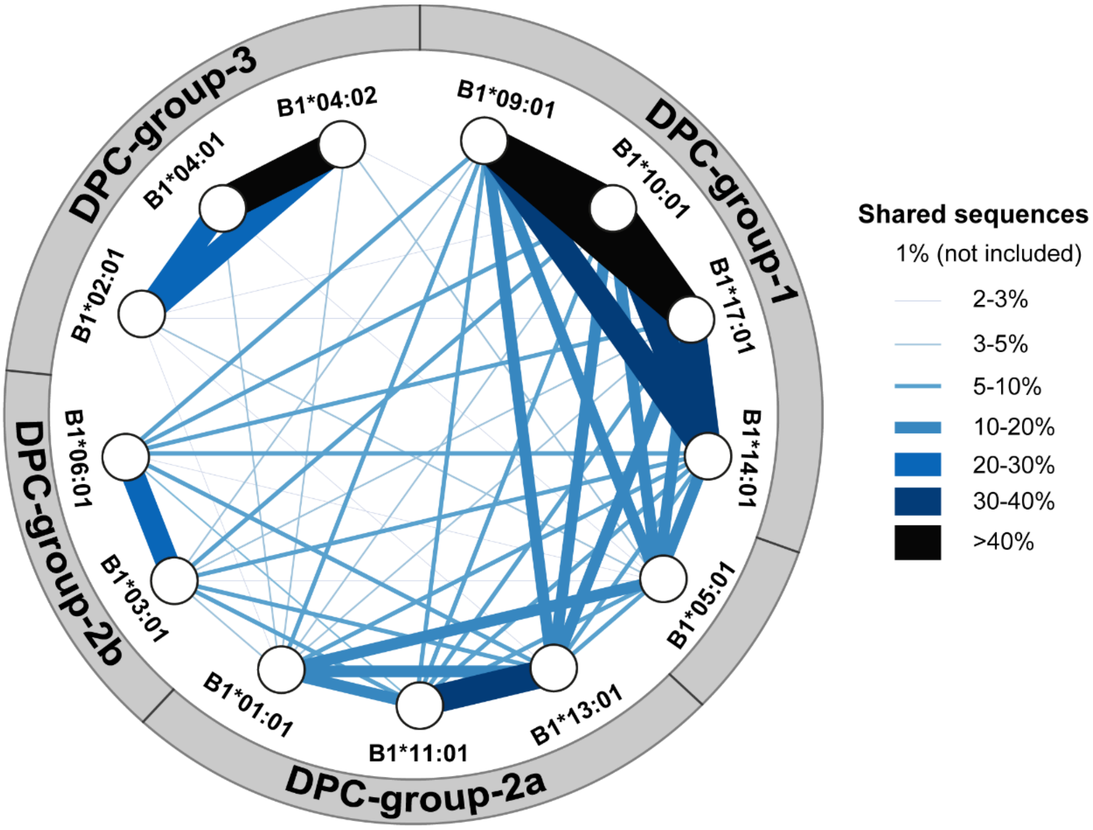

Supplementary Figure 3. Network of HLA-DP molecules. A network of HLA-DP molecules was generated based on the overlap in presented peptides. The length variants of peptide sequences were aligned to identify 10-30 amino acid long region sequences to include each distinct peptide only once in the quantifications. The percentage of overlap in presented peptides between the different HLA-DP molecules are illustrated by the thickness and color of the lines as indicated by the legend. The network shows the distinction of four major groups; group DPC-1 consisting of HLA-DPB1*09:01, HLA-DPB1*10:01, HLA-DPB1*17:01 and HLA-DPB1*14:01, group DPC-2A consisting of HLA-DPB1*13:01, HLA-DPB1*11:01 and HLA-DPB1*01:01, group DPC-2B consisting of HLA-DPB1*03:01 and HLA-DPB1*06:01, and finally group DPC-3 consisting of HLA-DPB1*02:01, HLA-DPB1*04:01 and HLA-DPB1*04:01.

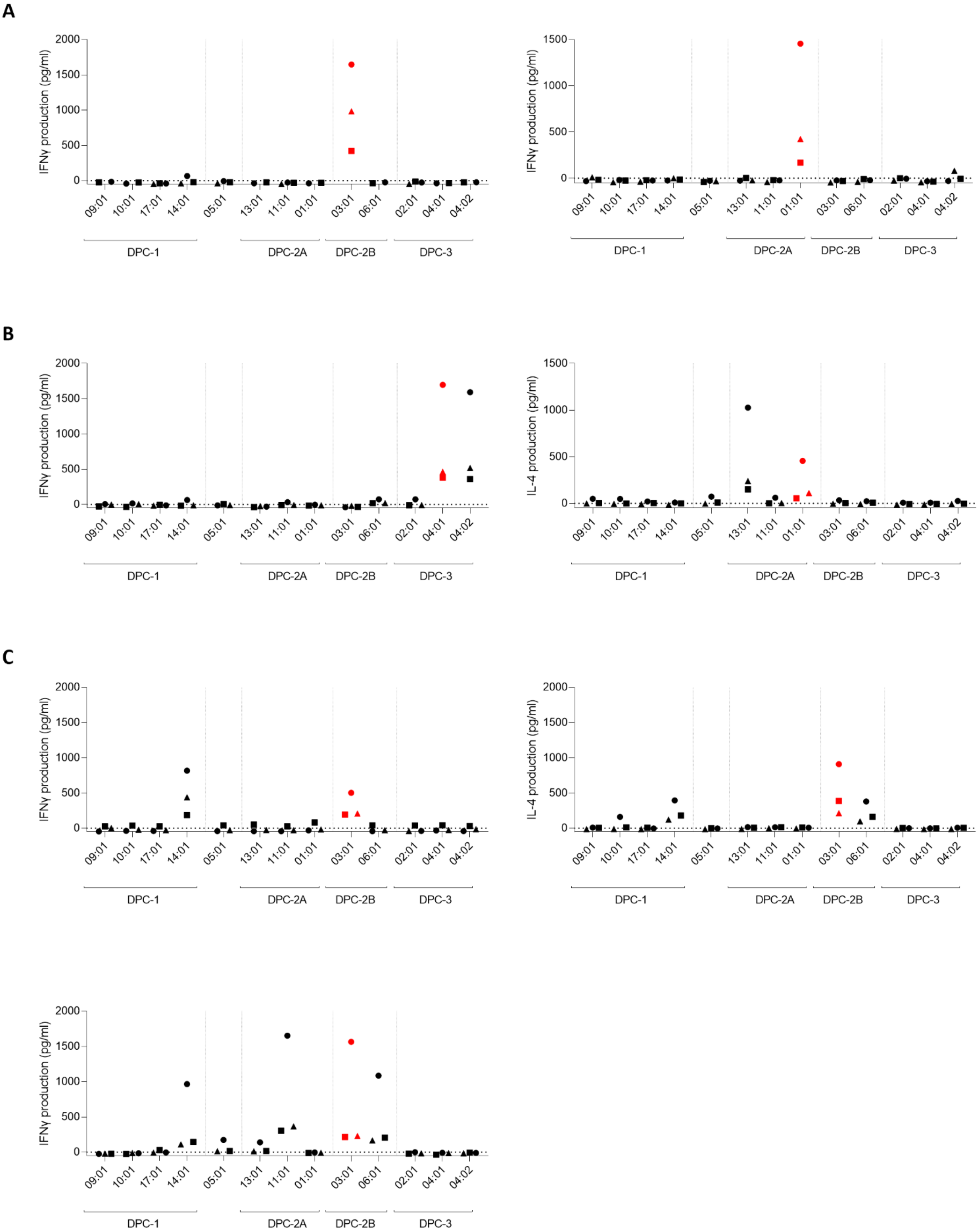
A
B
C
IFNγ production (pg/ml)
IL-4 production (pg/ml)
0
500
1000
1500
2000
09:01
10:01
17:01
14:01
05:01
13:01
11:01
01:01
03:01
06:01
02:01
04:01
04:02
DPC-1
DPC-2A
DPC-2B
DPC-3

Supplementary Figure 4. T-cell clones with cross-reactivity against HLA-DP molecules from the same or different DPC groups. The reactivity of allo-HLA-DP-directed CD4 T-cell clones after overnight stimulation with the panel of HLA-DP-transduced K562 cell lines. The concentration of IFNγ or IL-4 was measured in the supernatants by using ELISA. **(A)** CD4 T-cell clones with reactivity against single target HLA-DPB molecule. **(B)** CD4 T-cell clones with reactivity against the target HLA-DP molecule (red symbols) and cross-reactivity against a second HLA-DP molecule from the same DPC group. **(C)** CD4 T-cell clones with cross-reactivity against multiple HLA-DP molecules within the same DPC group as the target HLA-DP molecule, but also against HLA-DP molecules from another DPC group. The red colored symbols are the measurements after stimulation with the K562 expressing the target HLA-DP molecule. Each symbol represents one experiment.
